# Radionuclide Delivery Strategies in Tumor Treatment: A Systematic Review

**DOI:** 10.3390/cimb44080225

**Published:** 2022-07-22

**Authors:** Giulia Poletto, Diego Cecchin, Paola Bartoletti, Francesca Venturini, Nicola Realdon, Laura Evangelista

**Affiliations:** 1 Nuclear Medicine Unit, Department of Medicine DIMED, University of Padua, 35128 Padua, Italy; diego.cecchin@unipd.it (D.C.); paolabart23@gmail.com (P.B.); laura.evangelista@unipd.it (L.E.); 2 Pharmacy Unit, Padova University Hospital, 35128 Padova, Italy; francesca.venturini@aopd.veneto.it; 3 Department of Pharmaceutical and Pharmacological Sciences, University of Padua, 35131 Padua, Italy; nicola.realdon@unipd.it

**Keywords:** targeted radionuclide therapy, liposomes, avidin–biotin, docking

## Abstract

The aim of this review was to assess recent progress in targeted radionuclide tumor therapy, focusing on the best delivery strategies. A literature search was conducted in PubMed, Web of Science, and Scopus using the terms “radionuclides”, “liposomes”, “avidin–biotin interaction”, “theranostic”, and “molecular docking”. The 10 year filter was applied, except for the avidin–biotin interaction. Data were retrieved from both preclinical and clinical settings. Three targeting strategies were considered: pretargeting, liposomes, and ligands. Pretargeting can be achieved by exploiting the avidin–biotin interaction. This strategy seems very promising, although it has been investigated mainly in resectable tumors. Radiolabeled liposomes have attracted new interest as probes to identify the most suitable patients for treatment with liposomal formulations of common chemotherapeutics. The use of ligands for the delivery of radiotherapeutics to a specific target is still the most appealing strategy for treating tumors. The most appropriate ligand can be identified by virtually simulating its interaction with the receptor. All strategies showed great potential for use in targeted radionuclide therapy, but they also have numerous drawbacks. The most promising option is probably the one based on the use of new ligands.

## 1. Introduction

The goal of cancer therapy is to target and destroy tumor cells without damaging healthy tissues, but chemotherapy and external beam radiotherapy (EBRT)—the main alternatives to surgical treatment—are often nonspecific or associated with toxicity [1]. Many efforts have consequently been made to find ways to deliver cancer treatments more precisely. One example is targeted radionuclide therapy (TRT), a method whereby radionuclides are carried to tumor cells by molecules with a high affinity for the target [2]. Delivering radionuclides more effectively brings many advantages. For a start, the dose of isotope administered can be modulated, reducing patients’ exposure to radiation and the costs of the treatment. Second, a high affinity for the target can reduce background activity, thereby improving imaging quality and drug tolerability. Third, the opportunity to obtain information about a drug’s biodistribution using a diagnostic agent enables the planning of patient-specific treatments. To obtain the abovementioned advantages, the tracer is administered, at first, labeled with positron or γ-emitting radionuclides (e.g., ^18^F, ^68^Ga, or ^111^In) to see the areas where the tracer is deposited, identify any off-target uptake [3], and predict the dose absorbed by the tumor. Then the same tracer is administered again after labeling with β-emitting radionuclides (e.g., ^90^Y, ^177^Lu, or ^131^I).

Alpha-emitting radionuclides and Auger electron emitters can also be used for therapy, but β-emitting radionuclides are considered ideal for treating large tumors. This is because their long-range radiation can affect neighboring cells, as well as those being targeted (crossfire effect). Alpha-emitters are short-range, high-energy emitters more suitable for treating micrometastases and blood or bone marrow malignancies. Auger electron emitters are better suited to targeting single cells [1].

The choice of radiopharmaceutical agent is of primary importance in TRT. Carrier molecules should have a high affinity and specificity for the target, they should not be toxic or immunogenic, they should be stable before and after administration, they should be capable of binding a variety of radionuclides effectively, and they should be readily available at low cost [2].

Monoclonal antibodies (MoAbs) are among the most often used targeting agents because they can recognize a specific target and be bound directly to a radionuclide. They also have several drawbacks, however, such as a large size and slow kinetics [2]. The fact that some radionuclides decay rapidly has made it necessary to reduce the circulation time of MoAbs, and this has prompted the development of engineered antibody fragments, such as single-domain antibodies, diabodies, minibodies, protein scaffolds, and more complex specific antibodies [4]. Smaller antibodies have better pharmacokinetics and a good tumor penetration. They also have dimensions below the renal filtration cutoff; hence, they can be cleared through the kidneys, which are less radioresistant than the liver (the main site of MoAb accumulation) [4].

The high expression of peptide receptors on the surface of tumors means that peptide analogs are also good targeting agents [2]. Somatostatin receptors have been used as targets for over 20 years [5], especially for the treatment of neuroendocrine tumors. The most often used somatostatin analogs are dodecanetetraacetic acid phenylalanine-1 tyrosine 3-octreotide (DOTA-TOC) and dodecanetetraacetic acid tyrosine 3-octreotate (DOTA-TATE). They are labeled with ^90^Y and ^177^Lu for treatment purposes, and with ^68^Ga or ^111^In or ^99m^Tc for pretreatment imaging. The advantages of using peptide analogs include a well-established conjugation chemistry, an efficient penetration in solid tumors, and lower production costs [1].

Small molecules like hormones, steroids, and neurotransmitters that are internalized by specific receptors can also be used as targeting agents. An example is metaiodobenzylguanidine (MIBG), a structural analog of the neurotransmitter norepinephrine, which can be labeled with ^131^I or ^123^I for use in treating or imaging in patients with relapsing or refractory neuroblastoma, neuroendocrine tumors, or medullary thyroid cancers [1].

Despite this variety of promising targeting agents, the clinical efficiency of TRT remains low for solid tumors because the targeting agents become distributed mainly in the outer part of the tumor mass, with less radiation reaching the inner part [2].

In this systematic review, we analyze three possible strategies for delivering TRT in an effort to establish their efficacy. We focus on 1—the labeling of radionuclides on liposomes, 2—the adoption of a pretargeting strategy based on the avidin–biotin interaction, and 3—the feasibility of designing new ligands with a greater affinity for their receptors, by also virtually simulating the interaction (docking).

## 2. Materials and Methods

This review was conducted using the Preferred Reporting Items for Systematic Review and Meta-Analysis (PRISMA) approach. A comprehensive literature search was performed in the PubMed, Web of Science, and Scopus databases. Three different endpoints were considered for improving TRT: (a) the use of liposomes; (b) the avidin–biotin interaction; (c) molecular docking used to develop new ligands. The search strategies included the following combinations of words, adopted for each database: radionuclides AND delivery AND liposomes; radionuclides AND “liposomal doxorubicin”; liposomes AND theranostic AND radionuclides; radionuclides AND avidin AND biotin; “molecular docking” AND radionuclides.

The literature search and article selection were completed by two of the authors (G.P. and L.E.) from January to April 2022. One reviewer (G.P.) then ran a new search across the databases to check the reference lists in the selected studies and in review articles (excluded from the final analysis) to identify any additional papers covering the topics of interest.

Different filters were applied for the three areas of interest; for topics (a) and (c), all studies more than 10 years old were excluded, without discriminating between preclinical and clinical applications; for topic (b), only clinical applications of the avidin–biotin interaction were selected, with no cutoff for the year of publication (this approach was taken because clinical studies on the use of the avidin–biotin interaction for TRT were suddenly abandoned more than 10 years ago).

The quality of the clinical papers was assessed with a modified version of the Critical Appraisal Skills Program (CASP) checklist for cohort studies [https://casp-uk.b-cdn.net/wp-content/uploads/2018/03/CASP-Cohort-Study-Checklist-2018_fillable_form.pdf, access date: 25 May 2022]. This critical appraisal was performed by two reviewers (G.P. and L.E.), and any divergence of opinion was solved by discussion with a third author (D.C.).

## 3. Results

**Figure 1:**
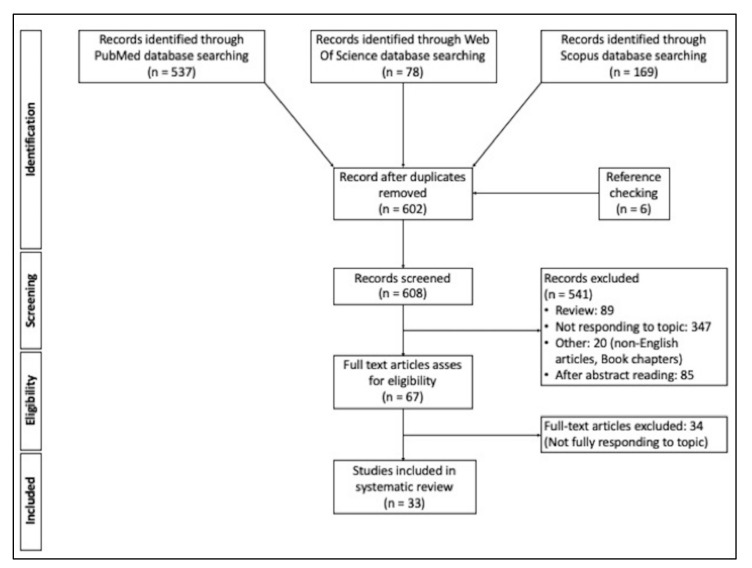
PRISMA flow diagram. Description of the search strategy and exclusion/inclusion criteria.

A total of 784 articles were found in the three electronic databases, and a further six articles emerged on checking the reference lists. All duplicates were removed, leaving 608 records. Then, all reviews and all articles not entirely consistent with our three areas of interest (if the delivery strategy was not studied with the intention of treating tumors, for instance) were excluded. The full texts of the remaining 67 articles were assessed for eligibility, leaving final total of 30 articles included in our review: 16 on topic (a), nine on topic (b), and five on topic (c). A summary of article selection process is shown in Figure 1.

The selected studies are discussed in detail in this section and are summarized in separate tables (Table 1, Table 2 and Table 3). 

The quality of the papers was only assessed for clinical studies (*n* = 12; Table S1); the main issue concerned the applicability of the results to clinical practice as most of the studies (*n* = 8) had an absence of clearly stated conclusions.

### 3.1. Liposomes

Liposomes are nanosized vesicles consisting of a lipid bilayer that can also contain cholesterol. They have an aqueous core and can be filled with either hydrophobic drugs (encapsulated in the bilayer) or hydrophilic drugs (encapsulated in the aqueous core). By means of a lipid chain, the main molecules (e.g., drugs and targeting agents) can also be linked to the membrane surface during liposome manufacture or via post-synthesis [6]. Embedding drugs in liposomes improves their properties, achieving a better biodistribution and a lower toxicity [7], and that is why these liposomal vesicles are often used for conventional drug delivery.

Liposomes were found to accumulate at tumor sites thanks to the enhanced permeability and retention (EPR) effect. They are easily taken up by the reticuloendothelial system (RES), however [7], consequently accumulating in organs such as the liver and spleen. To avoid their rapid clearance, polyethylene glycol (PEG) chains can be conjugated to the liposome surface to extend their circulation time and enhance their accumulation at tumor sites [7].

Studies on liposomes as drug delivery agents for use with radionuclides began in the last decade and led to the synthesis of a liposomal imaging tool called Vescan, which was never commercialized as it proved unable to detect tumors [36,37].

Attention has now shifted from the radiolabeling of empty liposomes to the radiolabeling of liposomal formulations of conventional chemotherapeutics. The aims of studies on this topic are to identify the pharmacokinetic (PK) properties of liposomal formulations once injected in vivo, and to establish which patients will better respond to this therapy.

Although liposomes are not a perfect example of a TRT, examining progress made in research on these vesicles can probably help us to better understand their potential future uses. We selected 16 articles on the radiolabeling of liposomal formulations of conventional chemotherapeutics: nine studies dealing with the pharmacokinetics of different liposomal formulations, and seven studies dealing with the patients’ different responses to the administration of liposomal formulations of chemotherapeutics. The content of these studies is summarized in Table 1.

The pharmacokinetic properties of liposomes may be influenced by the presence of a targeting agent on their surface. To give an example, Du and coworkers [13] synthesized liposomes functionalized with MoAbs against programmed cell death-1 (PD-1), a receptor selectively expressed in triple-negative breast cancer. These liposomes were then filled with doxorubicin (DOX) and dual-labeled with a fluorophore (IRDye800WC) and a radionuclide (^64^Cu). They proved better able to target and to treat the tumor due to the simultaneous effect of the MoAbs against PD-1 both as a targeting agent for liposomes and as an adjuvant immunotherapy for doxorubicin.

Alongside the presence of the targeting agent, the composition of a liposome may also influence its pharmacokinetic properties. Silva and coworkers [11] demonstrated that long-circulating, pH-sensitive liposomes (SpHL) containing [^99m^Tc] DOX accumulated more in the tumor and were less active in the spleen and liver than liposomes that were not pH-sensitive. That said, Monteiro and coworkers [16] noted that the presence of folate on the surface of SpHL (filled with paclitaxel) may lead to an even more sustained and higher tumor-to-muscle ratio than in the case of nonfunctionalized liposomes.

In addition to the presence of a targeting agent, other physical characteristics may enhance liposome delivery. For instance, Yang and coworkers [7] were able to obtain a good tumor brain delivery of their liposomal formulation of DOX, with a high tumor-to-contralateral brain ratio. They associated the presence of a targeting agent (AP-1, a peptide capable of binding IL-4 receptor) with the focused ultrasound technique, which enables a temporarily disruption of the blood–brain barrier. Reversible electroporation may also enhance delivery to the tumor, with or without any targeting agent on the liposome’s surface; this technique enhances vascular permeability, altering the EPR effect and, thus, leading to a greater liposome deposition at the tumor site [17].

To better study liposome distribution, the fluorescence technique can be associated with imaging, using positron emission tomography (PET), as in the earlier-mentioned work by Du et al. [13]. Li and coworkers [6] also succeeded in developing liposomes suitable for this application; their formulation could be labeled with the fluorophore IRDye-DSPE and the radionuclides ^99m^Tc, ^186/188^Re, or ^64^Cu thanks to the presence of DOTA on the liposome’s surface [6].

Luo and coworkers [15] demonstrated that adding porphyrin phospholipid to the liposome’s bilayer may also be useful for the development of liposomal vesicles suitable for multimodality imaging. 

Double radiolabeling is another way to obtain more information about the final target of both the liposome and the encapsulated drug. The feasibility of this technique was demonstrated by Lamichhane and coworkers [14], who labeled the liposome’s surface with ^111^In and the carboplatin derivative it encapsulated with ^18^F. More attention has also been paid in recent times to the search for new radiotracers compatible with the half-life of liposomes, and ^52^Mn has been identified as a suitable radionuclide for this purpose [18].

Pharmacokinetic studies have revealed a marked variability in liposome uptake by different tumors. This may be linked to the tumor’s mass, as Lin and coworkers [8] found in their study; small tumors showed growth inhibition with all the treatment regimens tested (liposomes containing chemotherapeutics and/or radionuclides), whereas the growth of large tumors was only significantly inhibited by a combination of chemo- and radiotherapy. It is not unusual to see a different liposome uptake in different patients with the same tumor or different tumors in the same patient. Tumor deposition is due mainly to the EPR effect, which could complicate pretreatment planning and hamper predictions regarding a patient’s prognosis [12]. 

The abovementioned studies on the pharmacokinetic properties of radiolabeled liposomes enabled tumor deposition and distribution to be quantified [9], making it possible to identify patients mostly likely to respond to a liposomal therapy. Before testing liposomes in humans, it was important to demonstrate the feasibility of radiolabeling preformed liposomal formulations. This was the goal of a study by Edmonds and coworkers [12], who successfully labeled liposomal formulations of drugs containing metal-binding motifs (e.g., doxorubicin and alendronate) with PET isotopes (e.g., ^89^Zr, ^52^Mn, and ^64^Cu) using metal ionophores (e.g., hydroxyquinoline).

The uptake of liposomal formulations can also be studied by recreating liposomes with the same lipid composition. This was achieved in vivo by Ito and coworkers [10], who synthesized liposomes with the same lipid composition as Doxil (a liposomal formulation of doxorubicin); they found a correlation between the therapeutic effect of Doxil and a histological factor associated with the EPR effect. 

Clinical studies on the biodistribution of liposomal formulations of chemotherapeutics in patients were made by Arietta and coworkers [19,20] and Lee and coworkers [21]. Arietta’s group examined the antitumor activity of a therapy combining liposomal doxorubicin (LD) with cisplatin in patients with malignant pleural mesothelioma. They labeled the LD with ^99m^Tc and found that patients who showed a ^99m^Tc-LD uptake of 75% or more had significantly better rates of response, progression-free survival, and overall survival than patients with uptake levels below 75%. The authors concluded that ^99m^Tc-LD uptake could be an important biomarker for use in assessing the results of therapy with LD and cisplatin [19,20]. Lee’s group radiolabeled MM-302, an HER2-targeted Doxil formulation, with ^64^Cu. After promising preliminary in vitro results [9], the liposomal vesicles were administered in humans [21], and the ^64^Cu-MM-302 uptake was found to vary considerably, both across multiple lesions in the same patient and across different patients. A high uptake in the liver was due to the physiological metabolism of liposomes. 

### 3.2. Avidin–Biotin Interaction

Avidin is a 66 kDa highly glycosylated, positively charged protein (isoelectric point~10) derived from egg white. It is tetrameric, and each monomer has a strong affinity for biotin (K_d_ = 10^−15^) [28,30,38].

**Figure 2:**
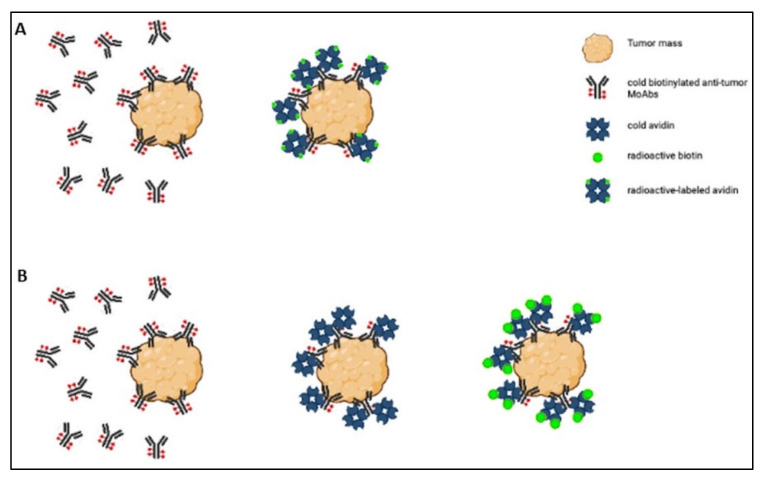
Scheme of the two-step (**A**) and three-step (**B**) protocols.

The strength of the avidin–biotin interaction is such that it is considered irreversible, and this explains why its applications have been the object of so much interest. For example, it has been studied in the sphere of tumor-targeted therapy for use in a pretargeting approach, which consists of delivering MoAbs and radionuclides separately. The radionuclide delivery is delayed until the MoAbs have reached the maximum tumor-to-normal tissue ratio [23], and the avidin–biotin interaction ensures the binding of the radiolabeled agent to the previously delivered antibody [22]. Two- or three-step protocols have been used in this setting (Figure 2). 

In the two-step protocol, the tumor is first targeted with cold biotinylated antitumor MoAbs, and then radioactive-labeled avidin is administered [23]. The three-step protocol involves (1) tumor pretargeting with cold biotinylated antitumor antibodies, (2) administering cold avidin to remove circulating biotinylated antibodies and ensure avidination of the biotinylated tumor-bound antibodies, and (3) labeling the tumor with radioactive biotin derivatives [22]. Both protocols have been tested in preclinical and clinical settings, but only the clinical studies are considered in this review. We selected nine articles that could be divided according to the type of tumor treated: three articles concerning various types of tumors, three articles concerning gliomas, and three articles dealing with breast cancer. A summary of the content of these articles is given in Table 2.

Paganelli and coworkers [22] first tested the feasibility of the three-step protocol in 20 patients with tumors expressing carcinoembryonic antigen (CEA). This preliminary study, conducted with ^111^In, revealed the advantages and disadvantages of the technique compared with the direct administration of radiolabeled MoAbs. The advantages included a drastically reduced background radioactivity, a well-preserved MoAb immunoreactivity (as autoradiolysis-induced damage to the MoAbs was avoided), and signal amplification. The main disadvantages were the need for repeated injections and the immunogenicity of avidin. 

Some years later, Cremonesi and coworkers [24] described the pharmacokinetic properties of the three-step protocol. The organs receiving the highest doses of radioactivity were the kidneys, liver, and urinary bladder, but the levels of renal, hepatic, or hematological toxicity were low. 

Paganelli and coworkers [23] also examined the application of the two-step protocol in the treatment of 15 patients with ovarian carcinoma. Here again, the high tumor-to-normal tissue ratio was highlighted as the main advantage of the method, and the repeated injections and use of streptavidin (an avidin analog) were identified as the main drawbacks.

After these first promising reports, the use of pretargeting strategies in tumor therapy spread and came to be applied to the treatment of malignant high-grade gliomas. In a phase I/II study, Paganelli and coworkers [25] used a three-step protocol to deliver 15 times more radioactivity to the sites of brain tumors than to critical organs (e.g., liver and kidneys). The treatment’s toxicity was consequently acceptable, with most of the activity not bound to the tumor eliminated in the first 24 h. The therapeutic benefit was evident in most patients (the tumor progressed no further in 52% of cases and shrank significantly in 25%), and the response persisted for more than 1 year in some patients. In this study, the only major drawback was again immunogenicity due to the administration of streptavidin. To solve this problem, the authors recommended using avidin modified with polyethylene glycol (PEG) molecules instead of streptavidin, as PEG can hide avidin from the immune system. Grana and coworkers [27] also tested a three-step protocol in the treatment of malignant gliomas, with promising results. The authors suggested that associating this technique with surgery, radiotherapy, and chemotherapy might increase the life expectancy of patients with high-grade gliomas. When Paganelli and coworkers [26] applied the three-step protocol in the locoregional treatment of patients with high-grade gliomas, they reported an objective therapeutic response in many patients, along with an encouraging median overall survival. On the basis of the neurological toxicity observed, they identified 1.11 GBq as the maximum tolerated dose.

All these studies on the avidin–biotin interaction led Paganelli and coworkers to develop a new procedure called IART (intraoperative avidination for radionuclide therapy) for use in the treatment of breast cancer. This procedure consists of two main steps: (1) “avidination” of the anatomical area of the lesion directly after tumor resection; (2) intravenous injection of radiolabeled biotin to target the anatomical area of the tumor 1 day after surgery. Before the radiolabeled biotin is injected, the circulating avidin is removed by injecting an appropriate amount (20 mg) of biotinylated albumin [28,29,30]. The rationale behind this procedure is that the inflammatory reaction after surgery makes the breast tissue a cation exchanger, thus enabling avidin retention at the site affected for several days [28,29,30]. The first studies using IART generated information on the biodistribution of biotin, which was labeled with ^111^In via the DOTA chelator. The radiolabeled biotin uptake appeared to be fast and stable at the operated tumor site, with a rapid blood and renal clearance, as well as a consequently reduced toxicity. The doses absorbed by the most affected organs (bladder and kidneys) were well below the threshold doses reported in the literature [28,29]. A more recent, phase II study was performed by Paganelli and coworkers [30] to quantify the doses administered with IART. The biologically effective dose (BED) to the tumor bed was 21 Gy when a fixed activity of 3.7 GBq of ^90^Y-DOTA-biotin was injected. The authors judged that IART can consequently be considered as a boost to tumor treatment, especially in association with EBRT. They concluded that this technique may be applicable not only to any breast cancer amenable to conservative surgery, as well as to many other solid tumors such as those involving the bladder, prostate, and brain [28,29,30].

### 3.3. Docking

Another possible strategy for performing an accurate TRT is to use a ligand with a very high affinity for the target. To ensure the strongest and most specific interaction, the ligand can be designed ad hoc, according to the structure of other known ligands or to the ligand’s interaction with the receptor. One way to ascertain whether the ligand thus designed is active is to use docking, a virtual simulation of ligand–receptor binding. The simulation of the interaction returns a score that can be used to quantify the ligand’s ability to bind its receptor.

Our literature search revealed five recent studies on the application of docking in the development of a ligand suitable for radionuclide delivery. Table 3 summarizes the content of these studies. In three of the five articles, docking was used to select the ligands with the strongest interaction with the receptor, while it was used to justify the results obtained in the other two articles. Some studies dealt with imaging rather than therapeutic goals (as the use of docking in ligand development is relatively new, there has been too little time to assess all the ligands for therapeutic applications), but we include them here to better explain the docking method.

Yang and coworkers [31] designed several new ligands for prostate-specific membrane antigen (PSMA) and used docking to identify the most promising among them. They synthesized two series of ligands based on a carbamate structure; one contained the amino-pentanedioic acid (NPA) moiety, while the other contained the oxypentanedioic acid (OPA) moiety. Then, they used docking to test the interaction between the carbamate derivatives and the PSMA. Their results showed that the Lys-OPA carbamates were better ligands than the Lys-NPA carbamates. Two of the former showed a high target-selective uptake in tumor xenografts, and one of the two (4-bromo-2-[^18^F] fluorobenzoyllysine OPA carbamate) also had a rapid normal organ clearance, making it the most likely candidate for clinical application.

Somatostatin receptor 2 (SSTR2) was the object of efforts to develop new ligands for treating neuroendocrine tumors (NETs). This receptor is the target of the previously mentioned somatostatin analogs DOTA-TOC and DOTA-TATE, but the focus of attention has recently shifted to developing receptor antagonists because they can bind to a larger number of sites, leading to a higher tumor uptake [33]. With this in mind, Behnammanesh and coworkers [33] developed a series of SSTR2 antagonists and labeled them with ^177^Lu by means of the DOTA chelator. Docking analysis then helped the authors to identify the peptide with the most successful accommodation at the binding site of the receptor (the DOTA-peptide 2, DOTA-p-Cl-Phe-Cyclo(d-Cys-l-BzThi-d-Aph-Lys-Thr-Cys)-d-Tyr-NH_2_). The same peptide was subsequently synthesized and tested in vitro and in vivo; it showed a good stability, had suitable pharmacokinetic properties, and was able to reveal tumor lesions, making it a promising therapeutic agent for NETs.

Kurniawan and coworkers [32] examined the fibroblast growth factor receptor 2 (FGFR2) as a target for use in the diagnosis and treatment of melanoma. They identified a water-soluble porphyrin—5,10,15,20-tetrakis-[3,4-bis(carboxymethylenoxy) phenyl]porphyrin (T3,4BCPP)—as the starting moiety for the development of a new ligand, and then they modified the meso-substituent to improve its solubility in water and affinity for the target, make it quicker to localize to the target, and achieve a high target-to-background ratio. They labeled the resulting ligands with ^99m^ Tc and ^188^Re, and tests led to the identification of porphyrin substituted with imidazole and carboxylic acid (3,4-BCP) as the best ligand for melanoma therapy. Docking analysis identified two new ligands (cD_3,4_BCPMIP and cD_3,4_BCPIP) as potential candidates for FGFR2.

Sarhan and coworkers [34] used docking differently, as a method for validating their findings. They developed an inhibitor of cyclin-dependent kinase 4 (CDK4), which can be used for the treatment of various neoplasms due to the overexpression of this kinase in multiple tumor cells. They synthesized a series of coumarin-based compounds and identified the one with the greatest potentially cytotoxic activity (assessed on MTT assay) and binding stability (assessed by docking). The ligand thus developed was radiolabeled with ^131^I and tested in vitro and in vivo, confirming its potential as a chemotherapeutic or radiotherapeutic agent, and as a radio-imaging agent in patients with solid tumors.

Matalinska and coworkers [35] worked on developing a new ligand for the neurokine-1 receptor (NK1R) for use in the TRT of glioblastoma multiforme. They started from L732,138, a ligand with a high affinity for NK1R, and expanded its structure to create five series of compounds bearing an unprotected amino group, an N-ter-butyloxycarbonyl group or an *N*-acetyl group at the end of the N-terminus. The five compounds that showed the highest binding capability in vitro were then labeled with ^177^Lu using DOTA as a chelator and tested in further in vitro studies. Docking was ultimately used to rationalize some of the results.

## 4. Discussion

In this review, we tried to identify a starting point for a possible alternative strategy to MoAbs and peptides for ensuring the delivery of radionuclides to tumor sites in TRT.

**Figure 3:**
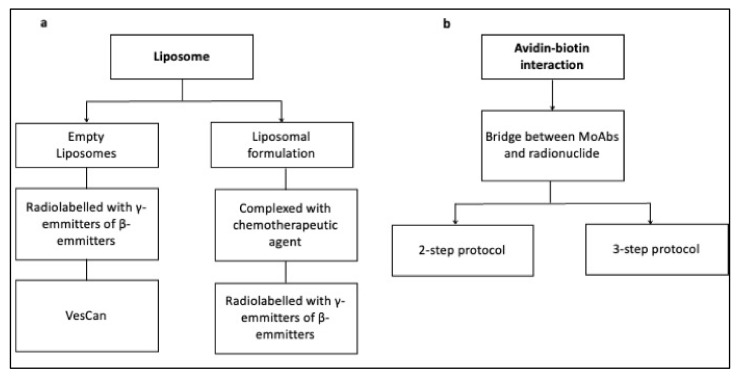
Schematic application of liposomes (**a**) and the avidin–biotin interaction (**b**) in clinical trials.

We considered three main categories: lipsomes, pretargeting, and new ligands. We focused only on liposomal nanoparticles because they have been the most often tested (also in clinical studies, Figure 3a). Other kind of nanoparticles have yet to be studied in human beings, despite promising preclinical results in their ability to deliver radionuclides. Moreover, the use of delivery systems can be advantageous in solving problems such as the resistance of some cancer cells. The use of copper sulfide (CuS) nanoparticles, indeed, has been proved to enhance, in vitro, the photothermal ablation (PTA) of cervical cancer cells. [39] Copper–cysteamine nanoparticles (Cu–Cy), moreover, can be used—combined with X-rays—to make an effective photodynamic therapy (PDT), thus enhancing the penetration depths of light and solving the problem of hypoxia associated with some kind of tumors [40,41,42,43,44].

As regards pretargeting uses, we only examined studies based on the avidin–biotin interaction. We consider this the most suitable pretargeting approach because of the strength of avidin–biotin binding, and because this technique has been amply tested for clinical applications (Figure 3b).

Judging from our quality assessment, the biggest weakness of the clinical studies on the use of liposomes and the avidin–biotin interaction for the purposes of cancer treatments lies in the small cohorts of patients enrolled. These cohorts were also sometimes not very clearly defined, and, in most articles, it was impossible to establish whether all the confounding factors were considered.

Liposomes have long been of great interest in the sphere of nuclear medicine, probably due to their versatility and easy synthesis. They were first studied for use in delivering radionuclides for imaging purposes (i.e., ^111^In), up until VesCan was developed and then unfortunately abandoned due to its weak ability to detect tumors. More recently, radiolabeled liposomes have been used mainly as probes in pharmacokinetic studies. Although this application seems far removed from the concept of a TRT, having the opportunity to visualize a therapeutic agent during its deposition in a tumor represents a huge advantage in cancer treatment, and a likely future application in nuclear medicine research. The practice of combining diagnostics and therapy—also called theranostic—has already gained substantial ground.

Studying the biodistribution of liposomal formulations of conventional chemotherapeutics brings another huge advantage: the chance to predict response to therapy. As some clinical studies showed, liposome deposition varies considerably between the same tumor in different patients and between different tumors in the same patient—with a consequent variability in their response to therapies. Such differences in liposome deposition are due mainly to the passive nature of their delivery, which takes advantage of the EPR effect. Many studies conducted in recent years to improve the deposition of liposomes filled with chemotherapeutics in tumors suffer have not gone beyond the preclinical stage; therefore, no data are available on the efficacy of such liposome deposition in humans. Given all the above considerations, we believe that the future for liposomes in TRT may be very promising. They will probably be tested for the simultaneous delivery of therapeutic radionuclides and chemotherapeutic agents with a view to their use in combined therapies; however, before that can happen, we need to solve the variability in their deposition in tumors, and their nonspecific uptake in the liver. Until now, very preliminary data are available about the labeling of liposomes with rhenium-186 [45,46]. However, as illustrated in Table 4, two clinical trials are ongoing for testing this therapeutic opportunity in patients with primary or secondary brain disease.

The second delivery strategy examined here concerns pretargeting. This approach was tested for the treatment of different kinds of cancer. Two main protocols involving two or three steps to deliver the radionuclide to the tumor site seemed to be effective, even in various types of solid tumor, and the clinical outcome for patients given these therapies seemed good. These studies also led to the development of IART, which has produced good results in the patients treated, especially when associated with EBRT. Unfortunately, the method has only been applied to breast cancer so far.

There are two main questions that arise from the studies considered here: (i) whether IART is an option for the treatment of tumors that are not surgically removable, and (ii) why studies on the use of pretargeting strategies for tumor therapy have been abandoned. The most recent study emerging from our literature search was conducted in 2010 and, to our knowledge, none have been conducted since. Given the strength of the avidin–biotin interaction, pretargeting certainly seems a good alternative to MoAbs and peptides for TRT, but more clarity is needed, including why the idea of using this technique for tumor treatment seems to have been shelved.

The third strategy for improving TRT considered here concerns the use of new ligands. As the discovery of new ligands can hardly be left to serendipity alone, we preferred to examine techniques for designing them. Among all the strategies available for use in designing a ligand, we considered docking, which enables the virtual simulation of a ligand’s interaction with its receptor. As emerged from our review, this method can be used both to identify ligands with the greatest affinity for a target and to justify previously obtained results. Docking, thus, represents a useful step in the design of new ligands, and its application could save time and money.

The biggest drawback of the studies that we analyzed lies in that none of the ligands developed have been tested in clinical studies, despite the promising results obtained in vitro (and, for some ligands, in vivo as well). In other words, we still cannot say for sure that the use of docking can lead to the development of a new ligand capable of carrying radionuclides straight to tumor sites. We do believe, however, that docking will be helpful in efforts to discover the so-called “magic bullet”. That said, we have to bear in mind that docking can only be used if we know the crystallographic structure of our ligand, which is not always so easy to establish.

## 5. Conclusions

Despite the small number of articles analyzed, this review provides some examples of what the future of TRT in nuclear medicine may hold. Liposomes are good delivery agents, but they are currently only used as probes for predicting patients’ response to a treatment with liposomal formulations of conventional chemotherapeutics. Pretargeting strategies relying on the avidin–biotin interaction are effective, but we need to know more about why they have not been further developed for use in treating cancer. Ligands are still among the most suitable candidates for improving TRT, but the discovery of new ones cannot be left to chance; they will probably be tailored to their receptors using methods such as docking.

## Figures and Tables

**Table 1:** (**a**) Preclinical studies on the use of radiolabeled liposomes in recent years. (**b**) Clinical studies on the use of radiolabeled liposomes in recent years.

(a)							
Reference	Year	Tracer	Type of Study	No. of Patients	Disease	Drug	Main Outcomes
**Shihong Li** [6]	2012	^99m^Tc ^64^Cu	Preclinical	None	Squamous cell carcinoma of head and neck xenograft	None	The simultaneous presence of a radionuclide and a fluorophore improves phamacokinetic studies
**Feng-Yi Yangh** [7]	2012	^111^In	Preclinical	None	Glioblastoma multiforme animal model	Doxorubicin	The association of focused ultrasound technique to the presence of a targeting agent on the liposome surface enhances their delivery to the brain
**Yi-Yu Lin** [8]	2013	^111^In	Preclinical	None	Colon carcinoma-bearing mouse model	Vinorelbine	The differences in tumor masses can be translated in different answer of the tumor to the therapy
**Helen Lee** [9]	2015	^64^Cu	Preclinical	None	Mammary tumor bearing mice	Doxorubicin	There is a heterogeneous distribution of liposomal drugs between different tumors
**Ken Ito** [10]	2015	^111^In	Preclinical	None	Human ovarian cancer xenograft	Doxorubicin	There is a correlation between the therapeutic effect of Doxil and histological factors associated with the EPR effect
**Juliana O. Silva** [11]	2016	^99m^Tc	Preclinical	None	Breast-tumor bearing mice	Doxorubicin	Long-circulating pH-sensitive liposomes show a higher tumor accumulation and a reduced spleen and liver activity compared to non-pH-sensitive liposomes
**Scott Edmonds** [12]	2016	^89^Zr ^52^Mn ^64^Cu	Preclinical	None	Metastatic mammary carcinoma mouse model	Alendronate Doxorubicin	Liposomes filled with drugs containing metal-binding motifs can be labeled with different isotopes by using metal ionophores like hydroxyquinoline
**Yang Du** [13]	2017	^64^Cu	Preclinical	None	Mammary tumor	Doxorubicin	The presence of MoAbs against PD-1 on the liposome surface enhance their targeting and therapy abilities. MoAbs against PD-1 work simultaneously as an adjuvant immunotherapy for doxorubicin chemotherapy.
**Nrottam Lamichhane** [14]	2017	^18^F ^111^In	Preclinical	None	None	Carboplatin	The labeling of both the liposome and the drug allows obtaining information on the destiny of the drug compared to the one of liposomes
**Dandan Luo** [15]	2018	^64^Cu	Preclinical	None	Mammary tumor bearing mice	Doxorubicin	The presence of a porphyrin phospholipid on the liposome bilayer may be useful for the development of nanoparticles suitable for imaging
**Monteiro LOF** [16]	2018	^99m^Tc	Preclinical	None	Human breast tumor xenograft	Paclitaxel	The presence of folate on the surface of SpHL leads to a higher tumor-to-muscle ratio than nonfunctionalized liposomes
**Govindarajan Srimanthveeravalli** [17]	2018	^89^Zr	Preclinical	None	Pancreas tumor xenograft	None	Electroporation enhances the EPR effect and, thus, the tumor deposition
**Peter Gawne** [18]	2018	^52^Mn	Preclinical	None	None	Doxil (doxorubicin)	^52^Mn may be a more suitable radionuclide for pharmacokinetic studies on liposomes due to the half-life compatible with the one of liposomes
**(b)**							
**Reference**	**Year**	**Tracer**	**Type of Study**	**No. of Patients**	**Disease**	**Drug**	**Main Outcomes**
**Oscar Arrieta** [19]	2012	^99m^Tc	Clinical	38	Malignant pleural mesothelioma	Doxorubicin Cisplatin	The combination of liposomal doxorubicin and cisplatin is an active combination for malignant pleural mesothelioma treatment with acceptable toxicity
**Oscar Arrieta** [20]	2014	^99m^Tc	Clinical	35	Malignant pleural mesothelioma	Doxorubicin Cisplatin	Patients that showed a ^99m^Tc-LD uptake of 75% or more had a statistically significant better response compared with those having uptake levels less than 75%
**Helen Lee** [21]	2017	^64^Cu	Clinical	19	HER-2-positive metastatic breast cancer	Doxorubicin	A variable ^64^Cu-MM-302 uptake was observed both across lesions within a patient and across patients; in patients with multiple lesions, not all of them had the same level of uptake

**Table 2:** Summary of clinical studies on pretargeting taking advantage of avidin–biotin interaction.

Reference	Year	Tracer	Type of Study	No. of Patients	Disease	Main Outcomes
**Giovanni Paganelli** [22]	1991	^111^In	Clinical	20	Different tumor types with increased circulating CEA	The advantages of the three-step protocol are the drastic reduction in the background radioactivity, the preservation of MoAb immunoreactivity, and the signal amplification The disadvantages are the need of repeated injections and the immunogenicity of avidin
**Giovanni Paganelli** [23]	1992	^111^In	Clinical	15	Ovarian cancer	The major advantage of the two-step protocol is the high tumor-to-nontumor ratio The major drawbacks are the repeated injections and the use of streptavidin
**Marta Cremonesi** [24]	1999	^90^Y ^111^In	Clinical	24	Different tumor types	Organs receiving the highest doses of radioactivity are the kidneys, the liver, and the urinary bladder
**Giovanni Paganelli** [25]	1999	^90^Y ^111^In	Clinical	48	Glioma (grade III or IV)	The application of the three-step protocol showed an evident therapeutic effect in most of the patients The only major drawback is the immunogenicity due to streptavidin
**Giovanni Paganelli** [26]	2001	^90^Y	Clinical	24	Anaplastic astrocytoma and glioblastoma	The three-step protocol can be used also for a locoregional treatment of gliomas; the maximum tolerated dose is 1.11 GBq
**Chiara Grana** [27]	2002	^90^Y	Clinical	37	High-grade glioma (grade III glioma and glioblastoma)	The three-step protocol can have an important role as adjuvant treatment in high-grade gliomas, due to its interference with progression, thus prolonging time to relapse and overall survival
**Giovanni Paganelli** [28]	2007	^111^In	Clinical	11	Breast cancer	The uptake of radiolabeled biotin appears fast and stable at the operated tumor site
**Giovanni Paganelli** [29]	2007	^111^In ^90^Y	Clinical	15	Breast cancer	There is an objective response to IART, with pain remission; IART can be used in any breast cancer amenable to surgery
**Giovanni Paganelli** [30]	2010	^111^In ^90^Y	Clinical	35	Breast cancer	IART provides a partial irradiation therapy immediately after surgery and shortens conventional EBRT

**Table 3:** Summary of studies on docking application for new ligand development.

Reference	Year	Tracer	Type of Study	No. of Patients	Disease	Target	Main Outcomes
**Xing Yang** [31]	2016	^18^F	Preclinical	None	Prostate cancer	PSMA	Lys-OPA-carbamates are more potent ligands for PSMA than the Lys-NPA carbamates 4-Bromo-2-[^18^F] fluorobenzoyllysine OPA carbamate is the candidate suitable for clinical tests
**Fransiska Kurniawan** [32]	2018	None	Preclinical	None	None	FGFR2	Porphyrin substituted with imidazole and carboxylic acid (3,4-BCP) is the starting point for the development of two new suitable ligand for melanoma therapy
**Hossein Behanammanesh** [33]	2020	^177^Lu	Preclinical	None	Neuroendocrine tumors	SSTR2	The authors were able to design a peptide with promising therapeutic properties for NETs
**Mona O. Sarhan** [34]	2021	^131^I	Preclinical	None	Ehrlich ascites carcinoma	CDK4	Coumarin can be used as the starting point for the development of a ligand able to bind CDK4
**Joanna Matalinsk** [35]	2022	^177^Lu	Preclinical	None	Glioblastoma multiforme	NK1R	Starting from L732,138, the authors were able to develop five ligands with high affinity for NK1R

**Table 4:** List of ongoing clinical trials with liposomes and avidin–biotin.

Number of Trial *	Status	Country	Type of Cancer	Type of Tracer
**NCT01906385**	Recruiting	USA	Glioma	Rhenium-186 liposomes
**NCT05034497**	Recruiting	USA	Leptomeningeal metastases	Rhenium-186 liposomes

* ClinicalTrials.gov.

## Data Availability

Not applicable here.

## Supplementary Materials

The following supporting information can be downloaded at https://www.mdpi.com/article/10.3390/cimb44080225/s1: Table S1. Assessment of clinical studies using Critical Appraisal Skills Program (CASP) checklist for cohort studies.
