# Supplementary material for: Radionuclide Delivery Strategies in Tumor Treatment: A Systematic Review
Source: Curr Issues Mol Biol. 2022 Jul 22;44(8):3267–82. doi: 10.3390/cimb44080225 (PMC9332578; doi:10.3390/cimb44080225)
Supplement: Supplementary file 1 [file cimb-44-00225-s001.zip › cimb-1797434-supplementary.pdf]

Table S1. Critical Appraisal Skills Program (CASP) checklist.

**Reference**		**21**	**22**	**23**	**28**	**29**	**27**	**30**	**31**	**32**	**25**	**33**	**26**
1. Did the study address a clearly focused issue?	*GP*	Y	Y	Y	Y	Y	Y	Y	Y	Y	Y	Y	Y
	LE	Y	Y	Y	Y	Y	Y	Y	Y	Y	Y	Y	Y
	AG												
2. Was the cohort recuited in an acceptable way?	*GP*	Y	Y	Y	Y	Y	Y	Y	Y	Y	Y	Y	Y
	LE	y	Y	Y	CT	CT	Y	Y	Y	CT	Y	Y	Y
	AG				**CT**	**CT**				**CT**			
3. Was the exposure accurately measured to minimise bias?	*GP*	Y	Y	Y	Y	Y	Y	Y	Y	Y	Y	Y	Y
	LE	Y	Y	Y	Y	Y	Y	CT	Y	Y	Y	Y	N
	AG							**CT**					**N**
4. Was the outcome accurately measured to minimise bias?	*GP*	Y	Y	Y	Y	Y	Y	Y	Y	Y	Y	Y	Y
	LE	Y	Y	Y	Y	Y	Y	CT	Y	Y	Y	Y	N
	AG							**CT**					**N**
5a. Have the authors identified all important confounding factors?	*GP*	Y	Y	CT	CT	CT	CT	CT	CT	CT	CT	CT	CT
	LE	Y	Y	CT	CT	CT	CT	CT	CT	CT	CT	CT	N
	AG												**N**
5b. Have they taken account of the confounding factors in the design and/r analysis ?	*GP*	CT	CT	CT	CT	CT	CT	CT	CT	CT	CT	CT	CT
	LE	CT	Y	CT	CT	CT	CT	CT	CT	CT	CT	CT	N
	AG		**Y**										**N**
6a. Was the follow up of subjects complete enough?	*GP*	Y	Y	Y	Y	Y	Y	Y	Y	Y	CT	Y	Y
	LE	Y	Y	Y	Y	Y	Y	Y	Y	Y	CT	Y	Y
	AG												
6b. Was the follow up of subjects long enough?	*GP*	Y	Y	Y	Y	Y	Y	Y	Y	Y	CT	Y	Y
	LE	Y	Y	Y	Y	Y	Y	Y	Y	Y	CT	Y	Y
	AG												
7. What was the result of this study?	*GP*	CLR	CLR	CLR	CLR	CLR	CLR	CLR	CLR	CLR	CLR	CLR	CLR
	LE	CLR	CLR	CLR	CLR	CLR	CLR	CLR	CLR	CLR	CLR	CLR	CLR
	AG												

8. How precise are the results?	*GP*	HIG	MED	HIG	MED	HIG	MED	MED	LOW	LOW	HIG	HIG	MED
	LE	HIG	MED	HIG	MED	HIG	MED	MED	LOW	LOW	HIG	HIG	MED
	AG												
9. Do you believe the results?	*GP*	Y	Y	Y	Y	Y	Y	Y	Y	Y	Y	Y	Y
	LE	Y	Y	Y	Y	Y	Y	Y	Y	Y	Y	Y	Y
	AG												
10. Can the results be applied to the local population?	*GP*	CT	CT	CT	CT	CT	CT	CT	CT	CT	CT	CT	Y
	LE	CT	CT	CT	CT	Y	CT	CT	CT	CT	CT	CT	Y
	AG					**Y**							
11. Do the results of this study fit with other available evidence?	*GP*	CT	Y	N	CT	N	N	N	Y	CT	CT	Y	Y
	LE	CT	Y	N	N	N	N	N	Y	N	N	Y	Y
	AG				**N**					**N**	**N**		
12. What are the implications of this study for practice?	*GP*	CT	CT	CT	CT	CT	CT	Y	Y	Y	CT	CT	Y
	LE	CT	CT	CT	CT	CT	CT	Y	Y	Y	CT	CT	Y
	AG												

GP: Giulia Poletto; LE: Laura Evangelista; AG: agree; Y: yes; N: No, CT: can't tell; CLR: clear; MED: medium; HIG: high
